# A Service Evaluation and Improvement Project: Reducing Delays in Transfer of Patients From Psychiatric Intensive Care Unit (PICU) to Prison After Completion of Treatment

**DOI:** 10.1192/bjo.2024.506

**Published:** 2024-08-01

**Authors:** Shantala Satisha, Emily Pettifor

**Affiliations:** Willow Suite Psychiatric Intensive Care Unit, Littlebrook Hospital, Dartford, United Kingdom

## Abstract

**Aims:**

The project aims to reduce the delays in transferring prisoners back to prison after they have completed the treatment of their mental health disorder in our male PICU.

Hypothesis:

When prisoners are admitted to our PICU for treatment of their mental health condition, there is a delay in transfer to prison after completion of their treatment due to lack of clear protocol between the services. We expect this project to significantly reduce these delays by agreeing treatment goals and exit pathways prior to admission.

**Background:**

Our 12 bed male PICU accepts admissions from prison for patients meeting our admission criteria. With increased number of admissions from prison since 2020, we were experiencing delays in transferring the patients back to prison after completing their hospital treatment.

**Methods:**

Data was collected for all admissions from prison services to the male PICU ward since June 2020 to April 2023. We introduced a PICU-Prison Transfer Agreement form in October 2021 which had to be signed by the mental health team and the governor of the prison before the admission. The form asked for details of any pending court appearances, solicitors’ details, release date, list of staff to be invited for CPA and agreement to accept the patient back to their prison after completion of treatment.

**Results:**

There were 44 referrals in this time period of which 24 were admitted to PICU. Prior to introducing the PICU-Prison Transfer Agreement, there was an average of 22.5 days (range 19–30 days) delay in patients being transferred to prison after being deemed ready for transfer. After the intervention, the delay in transfer reduced to an average of 10 days (range 5–11 days). The number of patients experiencing delay in transfer to prison of more than 2 weeks decreased from 100% to 0%.

**Conclusion:**

In conclusion, the project shows that a simple intervention of introducing an agreement form prior to admission has reduced the delays in patients being discharged from PICU to prison. It has also improved the quality of care with additional information provided in the form. When we accepted an admission from prison outside our county, prior to admission, the out of area prison arranged for a local prison to sign the agreement to accept the patient on discharge from PICU. This has led to a closer working and effective communication between the PICU and the prison services.

